# Two new species of *Membranacea* Qin & Zhang from China (Hemiptera, Cicadellidae, Typhlocybinae, Empoascini)

**DOI:** 10.3897/zookeys.260.4560

**Published:** 2013-01-21

**Authors:** Xiaofei Yu, Maofa Yang

**Affiliations:** 1 Institute of Entomology, Guizhou University, Guiyang Guizhou, 550025, P. R. China; 2 Key Laboratory for Plant Pests Management of Mountainous Region in Guizhou Province,; 3 Guiyang Guizhou, 550025, P. R. China

**Keywords:** Auchenorrhyncha, leafhopper, taxonomy, morphology, description

## Abstract

Two new species of the empoascine leafhopper genus *Membranacea* Qin & Zhang are reported from China: *Membranacea hubeiensis* Yu & Yang, **sp. n.** and *Membranacea stenoprocessa* Yu & Yang, **sp. n.**. A key to distinguish all species of the genus is provided.

## Introduction

The Typhlocybinae genus *Membranacea* was established by Qin & Zhang in 2011 for three new species from China with *Membranacea spinata* as its type species. Here we describe two new species from China and provide a key for the separation of all species. The examined specimens in this study are deposited in the Institute of Entomology, Guizhou University, Guiyang, Guizhou, China (GUGC).

## Materials and methods

The methods and terminology follow Zhang (1990) except for the nomenclature of wing, for which we follow Dworakowska (1993). Male specimens were dissected with the MOTIC B1 SMS-168 SERIES. Figures were made using OLYMPUS CX41 and enhanced using Adobe Illustrator CS4. Pictures were taken with VHX-1000C and dealt with Adobe Illustrator CS4. The body length is measured from the apex of the head to the apex of the forewing.

## Results

### Genus Membranacea

Qin & Zhang

http://species-id.net/wiki/Membranacea

Membranacea Qin & Zhang, 2011, Zootaxa, 2923: 48–58.

#### Type species.

*Membranacea spinata* Qin & Zhang, 2011

#### Description.

**Figures 1–18.**
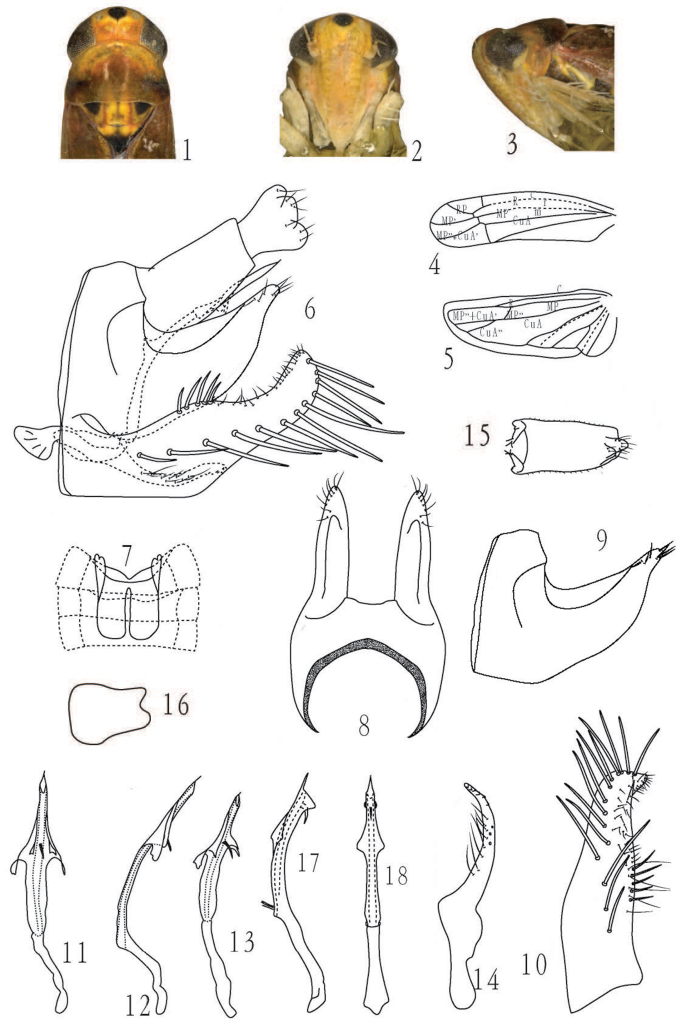
1–16 *Membranacea hubeiensis* Yu & Yang,sp. n., **1** head and thorax, dorsal view **2** face **3** head and thorax, lateral view **4** forewing **5** hindwing **6** male terminalia, lateral view **7** male abdominal apodemes **8** male pygofer, dorsal view **9** male pygofer, lateral view **10** subgenital plate, ventral view **11** aedeagus, ventral view **12** aedeagus, lateral view **13** aedeagus, lateroventral view **14** paramere, dorsal view **15** male anal tube, ventral view **16** connective **17–18** *Membranacea spinata* Qin & Zhang **17** aedeagus, lateral view **18** aedeagus, ventral view. Figs **17** and **18** from Qin, Liu & Zhang, 2011

**Figures 19–35.**
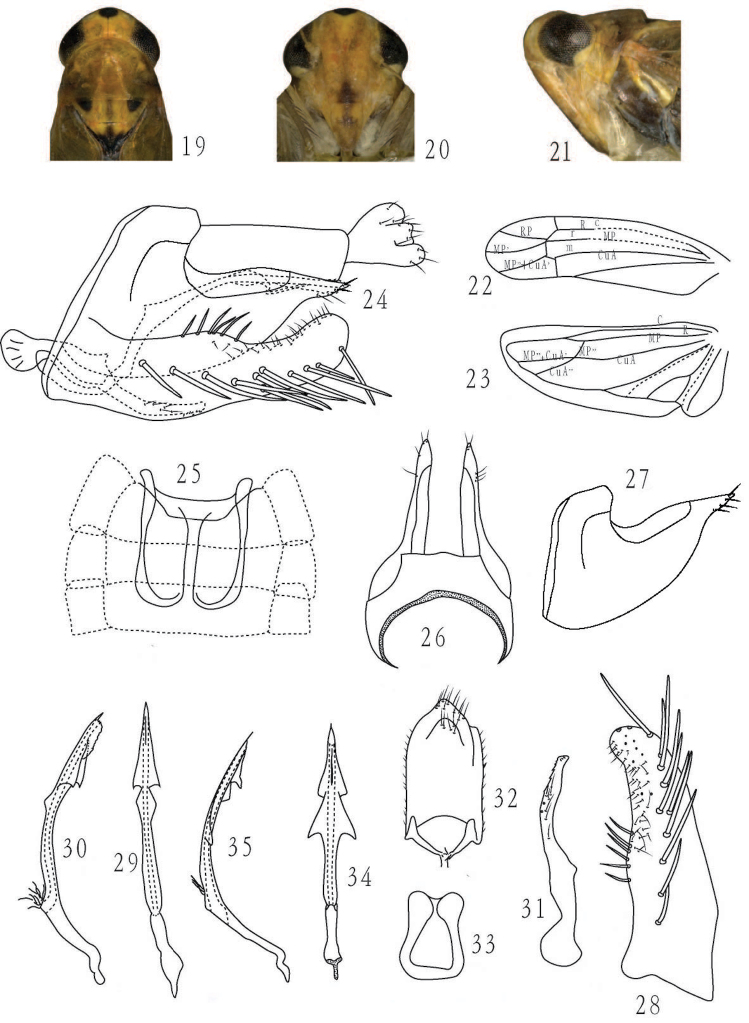
**19–33** *Membranacea stenoprocessa* Yu & Yang, sp. n., **19** head and thorax, dorsal view **20** face **21** head and thorax, lateral view **22** forewing **23** hindwing **24** male terminalia, lateral view **25** male abdominal apodemes **26** male pygofer, dorsal view **27** male pygofer, lateral view **28** subgenital plate, ventral view **29** aedeagus, ventral view **30** aedeagus, lateral view **31** paramere, dorsal view **32** male anal tube, ventral view **33** connective **34–35** *Membranacea plana* Qin & Zhang **34** aedeagus, ventral view **35** aedeagus, lateral view. Figs **34** and **35** from Qin, Liu & Zhang, 2011.

Body robust. Crown rounded at anterior margin, with a median black apical spot; coronal suture distinct, reaching anterior margin of vertex (Figs 1, 19). Ocellus present. Face elongated and convex in profile, lateral frontal suture present (Figs 2, 20). Pronotum wider and longer than crown (Figs 1, 19). Scutellum yellow with basolateral triangles and apex black; scutoscutellar sulcus distinct, not reaching lateral margin of scutellum (Figs 1, 19). Forewing rounded apically, apical cells less than one-third total length, RP and MP’ separated at base, both originated from r cell, MP’’+CuA’ from m cell, width of c cell equal with r cell (Figs 4, 22). Hindwing with CuA bifurcated, the branching point at or distad of coalescence of CuA with MP’’ (Figs 5, 23).

Male ventral abdominal apodemes well developed, rounded apically, reaching segment IV or V (Figs 7, 25). Male pygofer long, dorsal margin strongly concave, lightly sclerotized and less pigmented in basal 2/3 but more sclerotized apically, apex with few rigid microsetae; pygofer appendage absent (Figs 9, 27); dorsal bridge short (Figs 8, 26). Subgenital plate longer than pygofer (Figs 6, 24), basal broad, with an oblique line of macrosetae; outer margin slightly expanded at midlength and bearing few moderately long and stout setae forming the basal group (Figs 10, 28). Paramere shorter than pygofer, curved, narrowed from near midlength to apex, laterally with few setae, apex with teeth and sensory pits (Figs 14, 31). Aedeagal shaft elongated, curved posteriorly, with symmetrical flanges, gonopore apical on ventral surface; basal apodeme absent; preatrium developed (Figs 11, 12, 13, 17, 18, 29, 30, 34, 35). Connective with base broad, apex narrow (Figs 16, 33). Anal tube as in Figs 15, 32.

#### Distribution.

China (Guizhou, Hubei, Hunan, Shaanxi, Sichuan)

#### Remarks.

This genus is similar to *Alebroides* Matsumura, *Apheliona* Kirkaldy, *Nikkotettix* Matsumura, *Ghauriana* Thapa, *Matsumurama* Thapa, *Bhatasca* Dworakowska and *Luodianasca* Qin & Zhang in having veins MP’ and RP in the forewing arising from cell r and CuA in the hindwing branched apically. The genus differs from these genera in the pygofer having the dorsal margin strongly concave with a weekly sclerotized area, and from *Alebroides*, *Apheliona*, *Ghauriana*, *Matsumurama*, *Nikkotettix* in lacking the ventral pygofer appendage. The genus differs from *Bhatasca* in having the basal group setae of the subgenital plate located near the midlength of the dorsal margin and from *Luodianasca* in the absence of ventrally projecting anal tube processes, the abdominal apodemes well developed and the subgenital plate having setae in the basal group. It also differs from *Bhatasca* and *Luodianasca* in having the lateral macrosetae of the subgenital plate arranged in two rows submedially.

#### Key to species (male)

**Table:** 

1	Aedeagal shaft with two pairs of flanges (Figs 11, 18, 29, 34)	2
–	Aedeagal shaft with a pair of flanges	*Membranacea unijugata* Qin & Zhang
2	Aedeagal shaft with a ventral central spine (Figs 6, 11, 12, 13, 17, 18)	3
–	Aedeagal shaft without ventral central flange (Figs 29, 30, 34, 35)	4
3	Aedeagus with ventral central spine near apex (Figs 17, 18)	*Membranacea spinata* Qin & Zhang
–	Aedeagus with ventral central spine at or slightly beyond midlength of shaft (Figs 11, 13)	*Membranacea hubeiensis* Yu & Yang, sp. n.
4	Aedeagus with subapical flanges slightly broader than flanges at midlength of shaft (Figs 29, 30)	*Membranacea stenoprocessa* Yu & Yang, sp. n.
–	Aedeagus with subapical flanges distinctly narrower than flanges at midlength of shaft (Figs 34, 35)	*Membranacea plana* Qin & Zhang

### Membranacea hubeiensis

Yu & Yang sp. n.

urn:lsid:zoobank.org:act:798AFF17-4227-4ADE-9DB5-7DA56F59BA11

http://species-id.net/wiki/Membranacea_hubeiensis

Figs 1–16

#### Description.

Length, male 4.1–4.3mm.

General color reddish to yellowish orange. Eyes dark. Ocellus on anterior margin of crown, light brownish. Coronal suture brown margined with cream and with a yellowish brown spot on each side (Fig. 1). Face orange, paler on gena; some specimens with brownish stripe on anteclypeus (Figs 2, 3). Pronotum reddish orange centrally and posteriorly, with a median yellowish patch at anterior margin (Fig. 1). Scutellum yellow with basolateral triangles black margined with reddish orange; apex and sometimes a stripe medially, black (Fig. 1). Forewing reddish orange, semi-transparent in basal 2/3 and yellowish, hyaline in apical 1/3. Abdomen brownish. Legs yellow except midlength of hind tibia, brownish.

Ventral male abdominal apodemes broad, reaching segment IV or V, margins parallel or slightly divergent (Fig. 7). Pygofer with dorsal margin strongly concave, apex finger-like with few microsetae; dorsal bridge less than one-third of total length of pygofer (Figs 8, 9). Subgenital plate broad, with an oblique line of ca. 14 macrosetae and ca. 40 microsetae in 4 irregular rows; outer margin slightly expanded at midlength and bearing five moderately long and stout setae forming the basal group (Fig. 10). Paramere narrowed from near midlength to apex, curved laterally with ca. 9 fine setae, apex with teeth and sensory pits (Fig. 14). Aedeagal shaft elongated, curved posteriorly, subapically with a flange on each side and a narrower flange each side of a single central spine on ventral surface (Figs 11, 12, 13); preatrium nearly half length of shaft (Figs 11, 13). Connective with base broad, apex narrow, apical margin deeply emarginate (Fig. 16).

#### Type material.

Holotype, male, Houhe Natural Reserve, Wufeng City, Hubei Province, 27 July 2010, coll. Xiaofei Yu. Paratypes: 22 males, same data as holotype.

#### Etymology.

The new species is named after its type locality: Hubei.

#### Remarks.

The new species is similar to *Membranacea spinata* Qin & Zhang, 2011, but can be distinguished from the latter by the different configuration of the aedeagal processes (compare Figs 11–13 with 17, 18) and by the more pointed apex of the male pygofer (Fig. 9).

### Membranacea stenoprocessa

Yu & Yang sp. n.

urn:lsid:zoobank.org:act:56407D4D-CB37-4E0B-9089-AB10A6AD19DF

http://species-id.net/wiki/Membranacea_stenoprocessa

Figs 19–33

#### Description.

Length male 4.0–4.2mm.

Colour as for previous species but face dorsally and postclypeus yellowish orange, paler on anteclypeus, lorum, maxillary plate and gena, some specimens with a brownish stripe on anteclypeus and ventrally on postclypeus (Figs 19, 20, 21).

Ventral male abdominal apodemes broad, reaching the end of segment IV or V (Fig. 25). Pygofer with dorsal margin strongly concave, apex finger-like with few microsetae (Figs 26, 27). Subgenital plate broad, with ca. 12 macrosetae and ca. 42 irregular microsetae in 3 rows; outer margin slightly expanded at midlength and bearing 6 moderately long and stout setae (Fig. 28). Paramere narrowed from near midlength to apex, curved, laterally with ca. 7 fine setae, apex with teeth and sensory pits (Fig. 31). Aedeagal shaft elongated, curved posteriorly, with a flange on each side at midlength, a broader subapical flange on each side and a central flange on ventral surface extending to near apex, flange margins smooth to slightly dentate; preatrium less half length of shaft (Figs 29, 30). Connective with base broad, apex narrow, apical margin deeply emarginate (Fig. 33)

#### Type material.

Holotype: male, Houhe Natural Reserve, Wufeng City, Hubei Province, 27 July 2010, coll. Xiaofei Yu. Paratypes: 5 males, Xingdou mountain, Hubei Province, 4 August 2010, coll. Xiaofei Yu light; 1 male, Zhujia mountain, Guizhou Province, 25 July 2005, coll. Zaihua Yang; 1 male, Fanjing mountain, Guizhou Province, 21 September 2011, coll. Jiankun Long; 2 males, Longchang town, Xiuwen County, Guizhou Province, 8 July 2010, coll. Yinlin Mu.

#### Etymology.

The new species name alludes to the single narrowerventral flange on the aedeagal shaft.

#### Remarks.

The new species is similar to *Membranacea plana* Qin & Zhang, 2011, but differs from the latter in the more pointed apex of the pygofer and the slightly different shape of the aedeagal flanges with the lower dorsal pair slightly higher on the shaft (compare Figs 29, 30 with 34, 35). In one species of the genus (*Membranacea unijugata*) there is variability in the aedeagus suggesting that the new species could represent a variation of *Membranacea plana*. However, the differences seen between the two species are consistent in all the materials examined.

## Supplementary Material

XML Treatment for Membranacea

XML Treatment for Membranacea hubeiensis

XML Treatment for Membranacea stenoprocessa
